# Small Bowel Intussusception Caused by Metastatic Melanoma: A Case Report

**DOI:** 10.7759/cureus.5251

**Published:** 2019-07-27

**Authors:** Mohamed Ahmed, Husain Abbas, May Abdulsalam, Samir Johna, Rasha Saeed

**Affiliations:** 1 Surgery, University of California, Riverside, USA; 2 Advanced and Bariatric Surgery, Memorial Hospital, Jacksonville, USA; 3 Family Practice, Ibn Albaldi Hospital, Baghdad, IRQ; 4 Surgery, Loma Linda University School of Medicine, California, USA; 5 Surgery, Arrowhead Regional Medical Center, Fontana, USA

**Keywords:** melanoma, small bowel obstruction, metastatic disease, intussusception

## Abstract

Intussusception, as a rare cause of small bowel obstruction, can be secondary to benign or malignant pathology. Malignant lesions causing intussusception can be primary or metastatic lesions. Metastasis can occur many years later. We present a case of metastatic melanoma in a 69-year-old man as the underlying etiology of his intussusception. The patient had laparoscopic Roux-en-Y gastric bypass four years prior to his presentation and did recall excision of a skin melanoma at age 64. Laparoscopic or open surgical resection is the best therapeutic option in cases such as this.

## Introduction

One percent of small bowel obstruction in adults is caused by intussusception and is defined as the “invagination of a proximal bowel segment into the lumen of an adjacent distal segment” [1]. The lead points for intussusceptions are attributable to benign, malignant, or idiopathic causes [2]. Five percent of all gastrointestinal (GI) malignancies originate from the small bowel, and carcinoid is the most common, followed by adenocarcinomas, stromal tumors, and lymphomas [3]. Malignant melanoma are rare malignant tumors of the GI tract, and most of these tumors are secondary lesions of a primary location of the skin, anus, rectum, or eye [4].

## Case presentation

**Figure 1:**
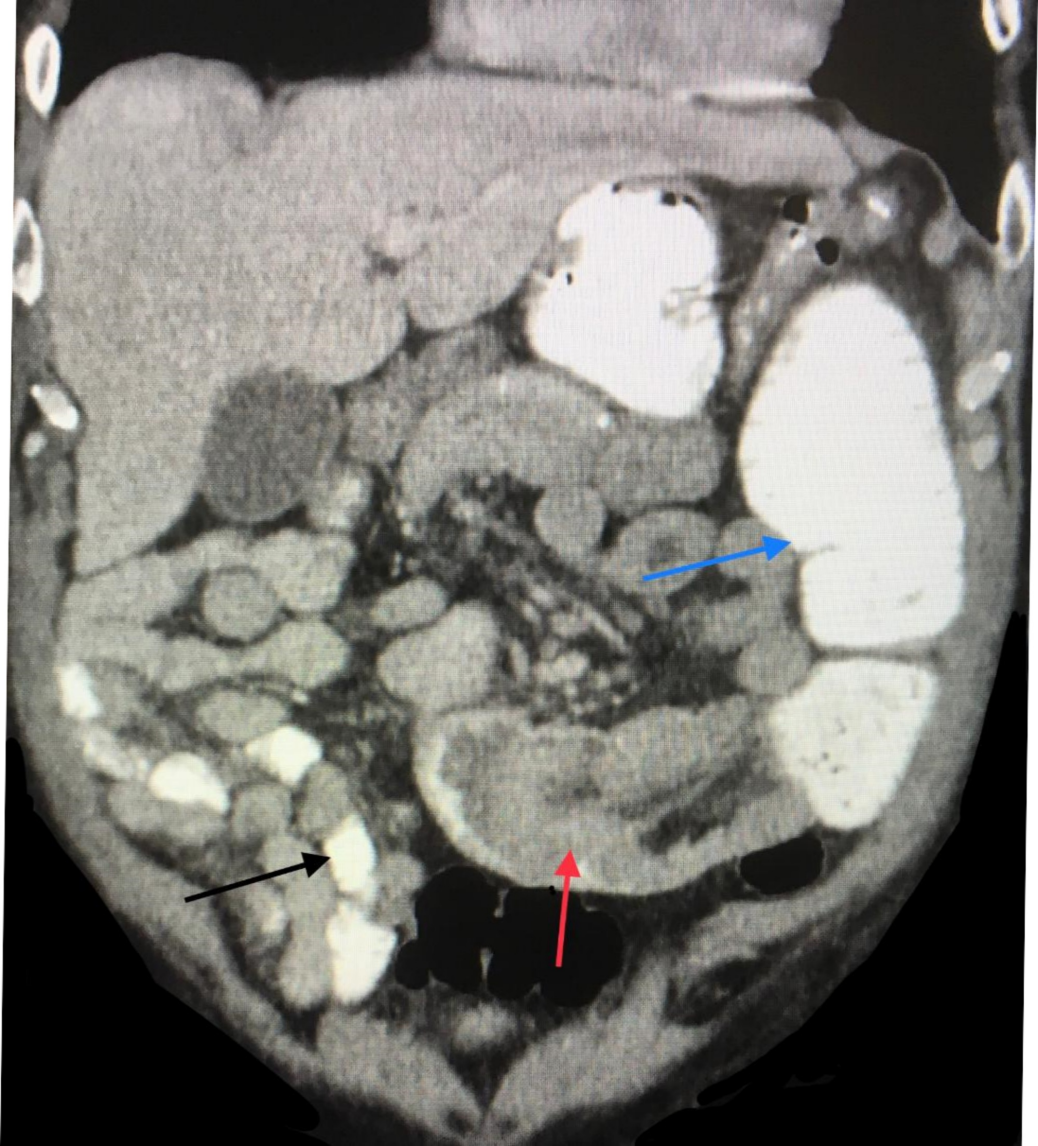
CT scan abdomen and pelvis Red arrow: jejuno-jejunal intussusception; Blue arrow: dilated bowel proximal to the obstruction; Black arrow: normal caliber bowel distal to the obstruction

A 69-year-old Caucasian man presented to our emergency room with a two-week history of worsening abdominal pain associated with nausea and vomiting. He had similar but less severe episodes multiple times, requiring hospitalization since his gastric bypass four years earlier. The findings of his abdominal and pelvic CT scan were consistent with small bowel obstruction secondary to jejunojejunal intussusception (Figure 1).

**Figure 2:**
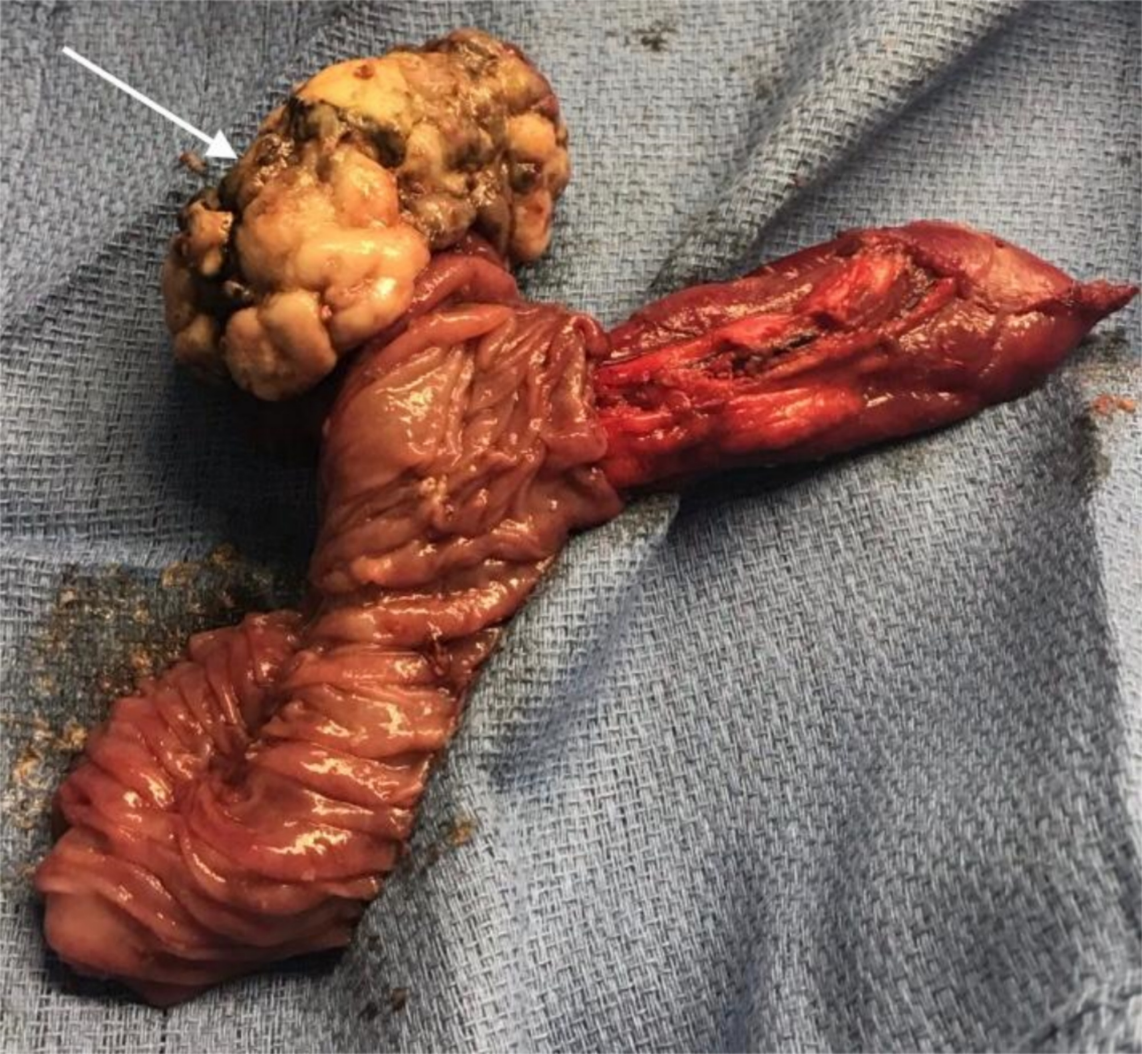
Resected Jejunum loop White arrow: tumor mass

The patient was taken to the operating room, and laparoscopic resection of the segment in question was performed. The intussusception leading point was consistent with a tumor mass (Figure 2).

**Figure 3:**
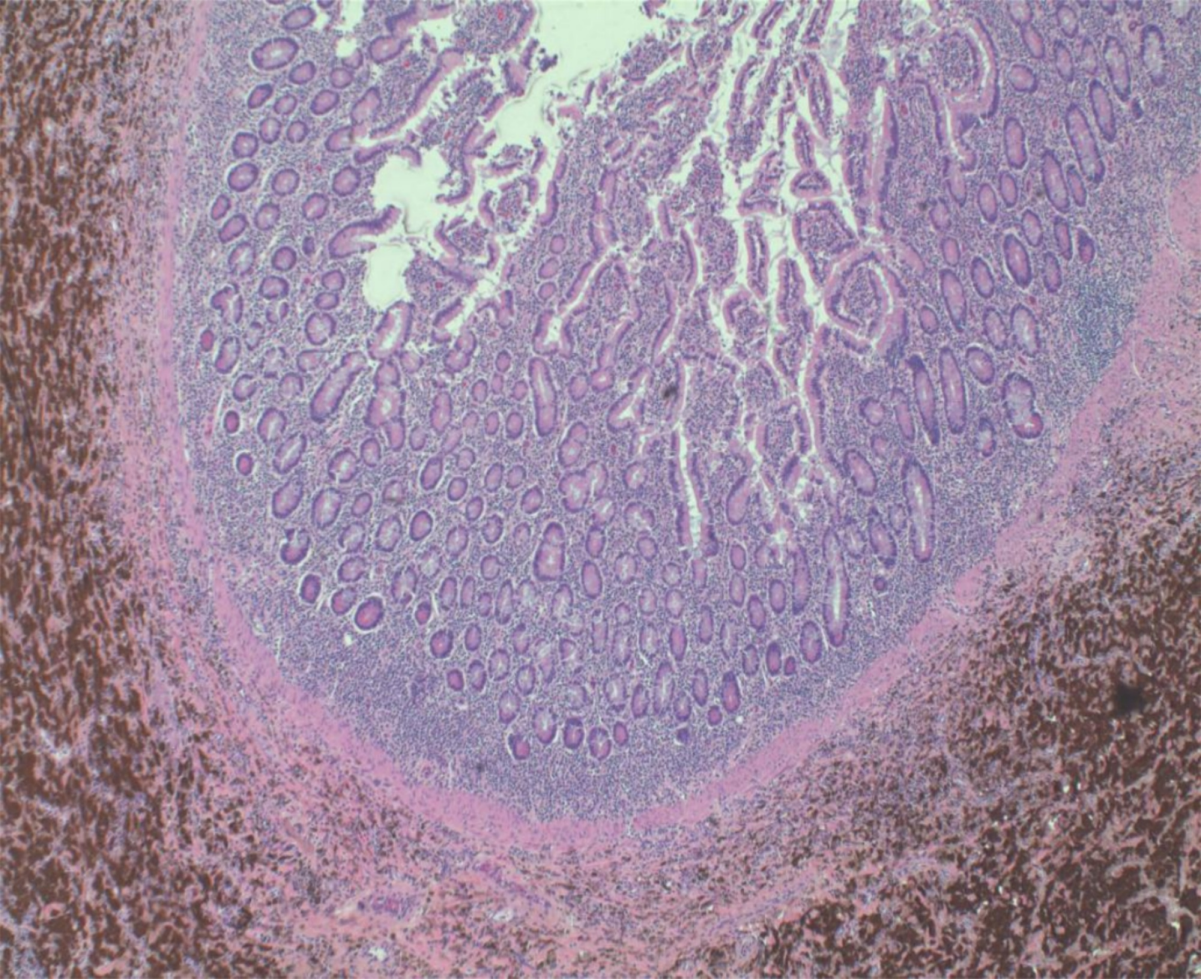
Histopathology Brown area: represents metastatic melanoma

The patient did well and was discharged from the hospital two days after admission. Pathological evaluation revealed a 6.5-cm malignant melanoma, and the immunohistochemistry analysis was positive for S100, melanoma antigen recognized by T-cells 1 (MART-1), and human melanoma back 45 (HMB45) and negative for iron stain and CD68, confirming the diagnosis (Figure 3).

The patient recalled a melanoma excision by his dermatologist six months prior to his gastric bypass.

## Discussion

Melanoma of the GI tract is rare and constitutes only 1% of all GI malignancies [5]. Most cases are due to metastasis from a primary cutaneous lesion, and few reports of primary melanoma of the small bowel exist in the literature. An autopsy study found that 60% of all patients with melanoma had intestinal metastasis, with the small intestine being involved in 50% of cases, the colon in 31%, and the ano-rectum in 25% [6]. Hintze et al. concluded that melanoma is the “most common extra-intestinal malignancy to metastasize to the [GI tract], and metastases can occur many years later” [6]. The higher incidence of metastasis to the small bowel may be due to its rich blood supply [7]. Currently, surgical resection of malignant melanoma of the GI tract performed with the laparoscopic or open technique is the treatment of choice [8-9]. Resection of melanoma metastases in the abdomen is associated with survival benefits, especially if abdominal metastases appear more than four years after the initial diagnosis and less than complete resection can also provide durable palliation [10].

## Conclusions

Intussusception in adults is a rare cause of small bowel obstruction and is usually caused by an underlying tumor, most often malignant. In our case, a metastatic melanoma from a skin lesion excised five years earlier was the cause. Laparoscopic or open surgical resection is the best therapeutic option.
